# The effect of climate change on depression in urban areas of western Iran

**DOI:** 10.1186/s13104-021-05565-0

**Published:** 2021-04-23

**Authors:** Hamed Abbasi

**Affiliations:** Department of Geography, Lorestan University, 6815144316 Khorramabad, Iran

**Keywords:** Climatic parameters, Trends, Depression, Linear regression, Variety of seasons

## Abstract

**Objective:**

Human is accustomed to climatic conditions of the environment where they are born and live throughout their lifetime. The aim of this study is to examine mood swings and depression caused by sudden climate changes that have not yet given the humans a chance to adapt.

**Results:**

Our results showed that depression could be affected by climate change and as a result, the behavior of climatic elements and trends has damaged mental health in the western regions of Iran. By investigating the trends and changes of climatic time series and their relationship with the rate of depression in urban areas of western Iran, it can be said that climate change is probably a mental health challenge for urban populations. Climate change is an important and worrying issue that makes the life difficult. Rapid climate changes in western Iran including rising air temperature, changes in precipitation, its regime, changes cloudiness and the amount of sunlight have a negative effects on health. The results showed that type of increasing or decreasing trend, as well as different climatic elements in various seasons did not have the same effect on the rate of depression in the studied areas.

## Introduction

One of the most important factors in controlling of human activities, is climatic factors. Climatic effects depend on several factors. The most important of them are geographical location and climatic elements of the regions. Climate change, temperature and rainfall affect various aspects of human life. One of the most important is physical and mental health. It not only affects physical health of the human beings but also the mental state of people to some extent.

Many researchers have studied the adverse effects of climate change on living environment and general health of the people in society [6, 4, 5, 8, 9, 14, 16, 23]. They have shown that unhealthy quality of life and people's health depends on climatic conditions.

There is an evidence to show that many behavioral illnesses and mental disorders, including major depression and bipolar disorder are affected by environmental and climatic conditions [1, 2, 10, 11, 17, 18, 21, 22].

Although researchers have suggested several potential causal factors for depression, including genetic factors, low socioeconomic status, and unpleasant life events, the impact of climate change remains relatively undiscovered. A special situation in the literature of this research is that climate change causes severe events such as intensification of heat storms, floods and coastal erosion, changes in soil quality, and these natural disasters may also disrupt the underlying economic and social structures of mental health. For example, climate change may alter favorable agricultural conditions, prevent agricultural practices, and ultimately lead to job insecurity and affect the progression of depression in residents of developing countries. When there is climate change such as in mountainous place where there is lots of snow in winter, people and the soil are used to the situation and when there is no snow fall people in that place suffer from lack of snow and the earth suffers too. The same thing happens when in some hot areas the climate gets hotter, thus causing a lot of problems and depression. In this study, we highlighted the role of climate change in the incidence and exacerbation of mental illness (depression and bipolar).

Diseases that are studied here are acute depression and bipolar disorder. The study of climatic parameters of temperature, precipitation and current climatic conditions on human behavior in Iran has been used by many researchers [20].

This study intended to identify mood changes and depressions caused by sudden climate change in urban areas of the Zagros Mountains and introduced the parameter that has the most impact.

## Main text

### Material and methods

#### Study area

**Fig. 1:**
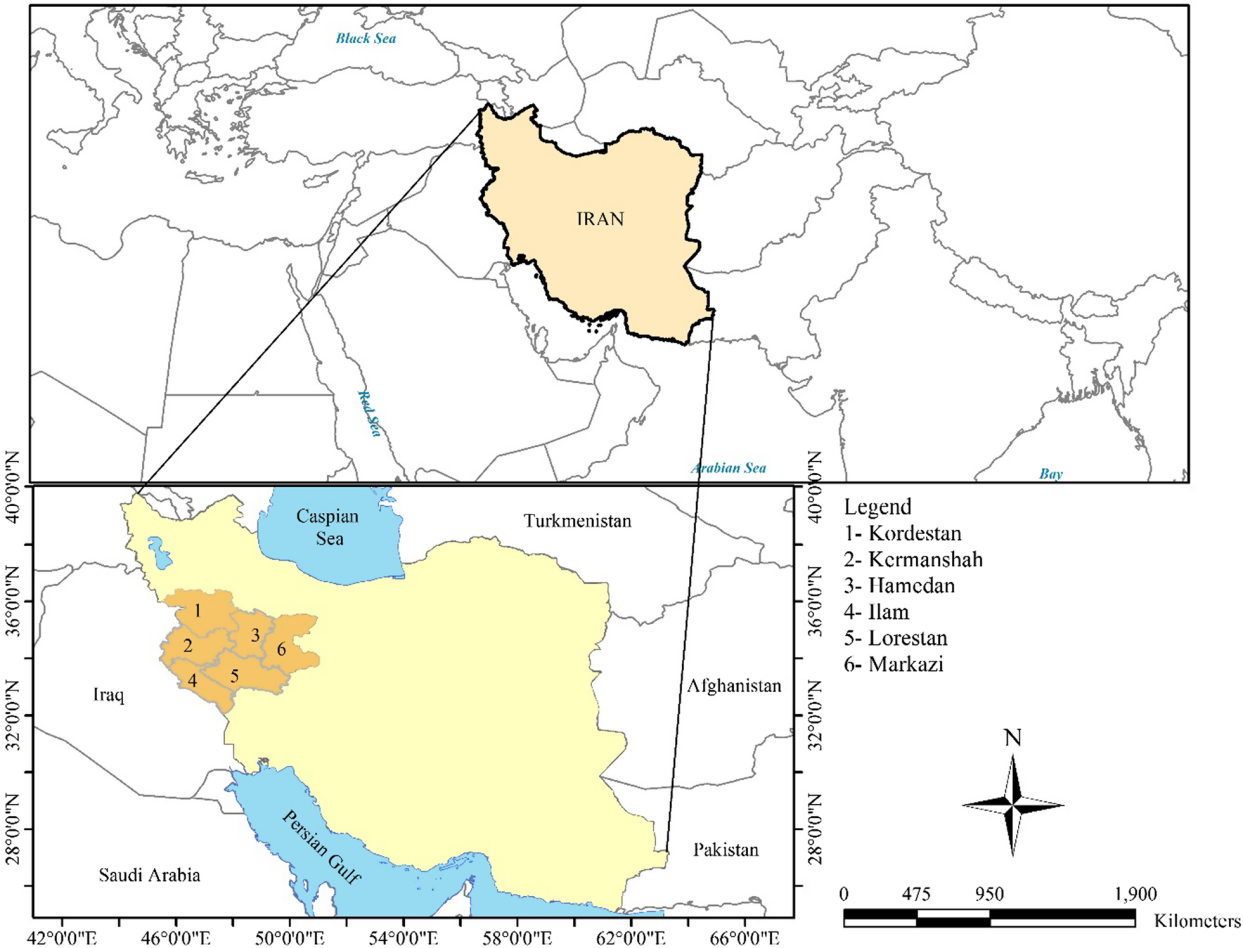
Geographical location of the study area (It was drawn by H.Abbasi using free QGIS software)

The studied areas are cities in western Iran, which are located in the Zagros Mountains. In most areas, the climate is cold and mountainous. In this regard, the statistics of synoptic stations in the region have been used. The geographical coordinates of the region are between 33°38′ to 35° 20′ N latitudes and between 46°26′ and 49°48′ E longitudes. Figure 1 illustrates the geographical location of the studied areas.

### Data collection and statistical analysis

Data were used and analyzed after some statistical tests such as run test, normal distribution and quality control. Data have a normal distribution and because of the independence of the residues, linear regression method was used. Regression method has been used in the studies such as [3, 13, 19, 20].

For assigning the relationship between climatic phenomena and mental disorders like depression, real data (minimum temperature, maximum temperature, precipitation, sunny hours, and cloudiness) were used monthly and quarterly for statistical period of 2004–2019. On the other hand, the data regarding depression were obtained based on the number of diagnoses from health centers. Due to the unavailability of statistics for depressed patients before 2004 in the target range, 15-year statistics have been used and we could not compare the statistics before 2004. Table 1 shows status of the trend of climatic parameters in the study cities.

**Table 1:** Status of the trend of climatic parameters stations of the study area

Station	Height (m)	Altitude	Latitude	Type of climate	Annual sunshine trend	Annual rainfall trend	Cloudiness (Okta) trend	max temperature trend	min temperature trend
Arak	1702	49° 48'	34°1'	cold semi-arid	3	12	0.1	1.8	0.5
Ilam	1360	46° 26'	33°38'	cold semi-arid	− 0.1	6	0.6	0.9	0.3
Kermanshah	1318	47°15'	34°35'	Cold semi-humid	4	− 2	0.4	1.2	0.25
Hamadan	1741	48°32'	34°52'	Very cold semi-arid	14	− 17	0.3	1.6	0.4
Borojerd	1629	48°45'	33°55'	Cold semi- humid	13	3	0.5	1.2	0.5
Khoram abad	1155	48°17'	33°26'	Cold semi- humid	11	− 11	0.5	1.3	0.6
Sanndaj	1373	47° 00'	35° 20'	cold semi-arid	10	13	0.1	0.8	0.3

### Measuring the effect of climate on depression

To model the count time series response using various covariates, negative binomial or Poisson time series model has been used. Assuming t as the unit of time, the method presented based on previous observations (${\alpha }_{r}$) as the *r*th fourth order correlation, the conditional mean *k* of the unit of time (θ) as the seasonal effect and the size of the estimated effect of the variables (${\beta }_{i}$). More details on the methodology, estimation and inference can be found elsewhere [7]. The Poisson time series model for the logarithm of the number of expected items ($\left(Log ({\lambda_t} )\right)$) can be written as follows:

$$Log\left( {\lambda_t} \right) = {\alpha_0} + \mathop \sum \limits_{r = 1}^R {\alpha_r}{Y_{t - r}} + \theta {Y_{t - k}} + \mathop \sum \limits_{i = 1}^p {\beta_i}{X_i}$$

The degree of correlation was examined based on the degree of correlation between the predictors and therefore some predictions were not included in the multiple models. The significance level was considered as 0.05.

## Results and discussion

Based on the results, it was found that increasing trend of the maximum and minimum temperature in summer causes more heat, depression, and exacerbation of disease. Table 2 shows the results of analyzing the relationship between trends of climatic elements and the two diseases (major depression and bipolar disorder). In winter, the increase in the temperature in the studied areas moderates winter cold in the Zagros heights. Therefore, this increase can lead to a reduction in depression, but since in most of the studied areas, parameters such as cloudiness are increased in winter, therefore these diseases are exacerbated in these areas.

**Table 2:** Results of analyzing the relationship between trends of climatic elements on the frequency of major depression and bipolar disorder

Response	Independent variable	Coefficient	P-value	*e*^Estimate^
Major depression				
	Max temperature	0.230	0.009	1.259
	Max temperature trend	0.442	0.006	0.843
	Min temperature trend	− 0.508	0.046	0.602
	Precipitation	0.004	0.018	0.604
	Mean temperature	− 0.242	0.012	0.785
	Mean temperature trend	0.12	0.007	1.606
	Seasonal effect	0.400	0.005	1.492
	Cloudiness (Okta) trend	0.642	0.008	0.943
	Shiny hours	0.007	0.003	1.2
	Shiny hours trend change	− 0.048	0.005	0.953
Bipolar				
	Max temperature trend	0.08	0.001	0.85
	Min temperature trend	0.006	0.008	0.992
	Precipitation trend	0.004	0.007	0.82
	Cloudiness (Okta) trend	0.442	0.007	1.143
	Shiny hours	0.002	0.028	1.002
	Shiny hours trend change	− 0.24	0.025	0.986

Climate parameters of cloudiness did not show a significant trend, but its trend in spring in this area was increasing, so that it had no effect on bipolar disorder but they led to acute depression. This increase was due to climate change and variability of precipitation regime in Iran [15]. In autumn, the amount of cloudiness and precipitation has decreased, which shows that in these areas, autumn has become drier than before. At the beginning of autumn it seems that it exacerbates depression, but dry air (until mid-autumn) has led to a reduction in depression in the region, which of course can be related to the beginning of autumn and the adjustment of temperatures in these areas. From semi-autumn, with the arrival of the cold season, the amount of cloudiness and precipitation increases with the arrival of western Mediterranean systems which have the greatest climatic effects in western Iran. By increasing the amount of cloudiness along with the decrease of sunny hours in winter, there is a positive effect on the rate of severe depression, bipolar disorder and the number of cases with these diseases increases as well.

Based on findings about depression in these cities, the increasing trend of spring clouds and the decreasing trend of sunny hours in winter had the greatest influence on the aggravation of depression among the climate change and trends studied.

The number of cloudy days is very low in the study area due to establishment of subtropical high-pressure summer. Therefore, frequency of depression in this season is related to the increase in the temperature and its increasing trend. In winter, with arrival of west wind systems of subtropical region in the studied areas, the number of cloudy days is increased. On the other hand, sunny hours are significantly reduced due to shortening of the day, therefore it can be said that cloudiness and reduction of sunny hours are the main climatic factors which are responsible for aggravation of depression in western region of Iran in cold season of the year. There is an important matter that the trend of climate change in depression leading to different consequences in various seasons .Obviously, sunny days in cold season of the year bring about more happiness in the studied areas, and on cloudy days with the decrease in sunlight, emotional disorders are intensified.

How climatic parameters, trends and their changes affect depression also depends on its persistence. Increasing of cloud cover is not the same in all studied areas. In semi-desert and inland areas such as Arak and Hamadan despite the increase cloudy days, rainfall has decreased. In more northern regions such as Sanandaj, due to the increasing of cloudy days the amount of rainfall has also increased. The different effects of rainfall behavior and sunny hours in different regions and times can be considered. The average number of sunny hours in all areas has been increasing. These increases together with the increase in rainfall in some areas indicates that heavy precipitation have been caused by climate change in these area.

Studying the impact of climate trends on bipolar disorder, it was found that this disease is less dependent on environmental and climatic conditions than depression. However, some seasonal trends such as decreasing trend of sunny hours and its changes in winter in some of the studied cities aggravate this disease while the trend of parameters such as cloudiness and spring temperature has little effect on its intensification.

The effect of increasing the maximum temperature of summer is also significant in exacerbating this disease. In other words, rising air temperature, intensification of summer heat in arid climate and long summer days in the studied areas cause dissatisfaction of patients with bipolar depression, therefore it seems that this disorder is more related to genetic factors. Climatic conditions and climate change have a lesser role in exacerbating the disease.

## Conclusions

Climate change influences many aspects of human activities; the most important of which is the health sector. According to careful observation of the environment, it can be found that climate change leads to different reactions in the human body and mind. Climate change seems to be an important factor leading to a decrease or increase in the stress and change in the humans҆ behavior. There is even a direct connection between these changes, trends and prevalence of some mental disorders which received less attention. Given the importance of this issue, the main objective of this research was identifying climate trends in urban areas in western Iran and investigating their effect on mental disorders, such as depression and bipolar disorder.

Studies conducted in the area showed that increasing the duration of sunshine in the cold season has a very important function in increasing people's satisfaction relative to their surroundings. Given that most people feel depressed and upset on cloudy and rainy days, it is necessary to examine the impact of climate and its changes on humans on a larger scale. This result confirms the studies of [8, 12, 24].

In recent years, the rate of depression in urban areas of Iran has increased because of various reasons, part of which can be due to environmental conditions and climate change. Since the rainfall systems enter Iran from the west, the western regions have more rain and more cloudy days. It seems that the effect of changes in rainfall regime and the behavior of rainy seasons is more important than changes in rainfall in these areas, but in the eastern regions due to higher altitude (urban area of Hamadan and Arak) and increasing drought coefficient and many differences in winter and summer temperatures (80 °C), trends and temperature changes were more important, and one of the consequences of this increasing trend of temperature is the increase and occurrence of depression in these areas.

Considering the direct effect of rain and sunny hours on depression, it can be said that heavy rainfall, seasonal changes, the behavior of rain and sunny hours directly play an important role in the occurrence of depression and light falling of the rain in these areas with semi-arid climates can have a direct effect on reducing of depression.

## Limitations

One of the main limitations of this study was the lack of long-term and sufficient data on depression in Iran.

In addition to the lack of data on depression, the most important matter and limitation in conducting this study was that some depressed patients didn't go to the medical centers to be registered for their illness, so it was hidden in this study.

## Data Availability

According to the memorandum No.14/20,008 dated 15/02/2020 (through Lorestan University); the data has been taken and analyzed. The data is available upon the request from the authors.
